# Periosteum: A Highly Underrated Tool in Dentistry

**DOI:** 10.1155/2012/717816

**Published:** 2011-09-25

**Authors:** Ajay Mahajan

**Affiliations:** Department of Periodontics, Himachal Pradesh Government Dental College and Hospital, Snowdown, Shimla 171001, India

## Abstract

The ultimate goal of any dental treatment is the regeneration of lost tissues and alveolar bone. Under the appropriate culture conditions, periosteal cells secrete extracellular matrix and form a membranous structure. The periosteum can be easily harvested from the patient's own oral cavity, where the resulting donor site wound is invisible. Owing to the above reasons, the periosteum offers a rich cell source for bone tissue engineering; hence, the regenerative potential of periosteum is immense. Although the use of periosteum as a regenerative tool has been extensive in general medical field, the regenerative potential of periosteum is highly underestimated in dentistry; therefore, the present paper reviews the current literature related to the regenerative potential of periosteum and gives an insight to the future use of periosteum in dentistry.

## 1. Introduction

Reconstruction of lost tissues is a long cherished goal in medical field. A lot of research has been done in the past, and still research is going on to explore tools and techniques for regeneration of lost tissues as a result of the disease process. The use of various grafts and recent tissue engineering techniques including stem cell research are testimony to the ever increasing need for most suitable treatment option to replace/repair lost tissues due to various pathologic processes. The use of autogenous periosteum in general medical treatment has been extensive and has shown promising results [1–3]; on the contrary in dentistry, the use of periosteum as a regenerative tool has been limited and highly underrated; therefore, the purpose of this paper is to highlight the current status of use of periosteum in dentistry as well as suggesting its future use in various treatment options related specifically to dental field. 

## 2. Periosteum: What Justifies Its Use?

**Figure 1:**
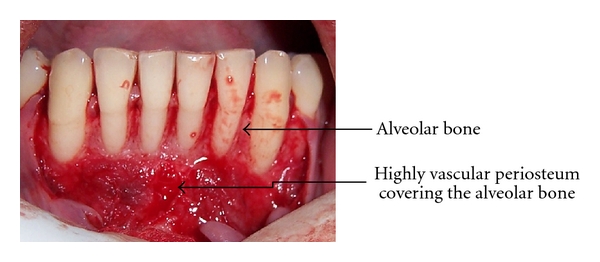
Highly vascular periosteum covering the alveolar bone.

**Figure 2:**
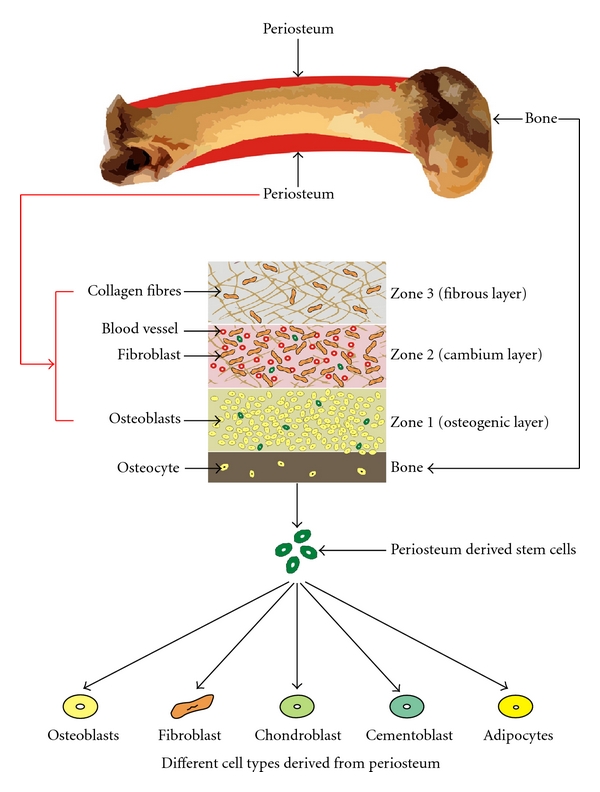
The three different Zones of periosteum; Zone 1 has an average thickness of 10–20 um consisting predominantly of osteoblasts; the majority of cells in Zone 2 are fibroblasts, with endothelial cells being most of the remainder. Zone 3 has the highest volume of collagen fibrils among all the three zones. The bottom of the figure shows regenerative capacity of the periosteum to form different cell types.

The periosteum is a highly vascular connective tissue sheath covering the external surface of all the bones except for sites of articulation and muscle attachment (Figure 1) [4]. The periosteum comprises of at least two layers, an inner cellular or cambium layer, and an outer fibrous layer [1]. The inner layer contains numerous osteoblasts and osteoprogenitor cells, and the outer layer is composed of dense collagen fiber, fibroblasts, and their progenitor cells [5]; osteogenic progenitor cells from the periosteal cambium layer may work with osteoblasts in initiating and driving the cell differentiation process of bone repair characterized by the development of the initial fracture callus and subsequent remodeling. Periosteum can be described as an osteoprogenitor cell containing bone envelope, capable of being activated to proliferate by trauma, tumors, and lymphocyte mitogens [6]. Research on the structure of periosteum has shown that it is made up of three discrete zones [7]. Zone 1 has an average thickness of 10–20 um consisting predominantly of osteoblasts representing 90% of cell population, while collagen fibrils comprise 15% of the volume. The majority of cells in zone 2 are fibroblasts, with endothelial cells being most of the remainder. Zone 3 has the highest volume of collagen fibrils and fibroblasts among all the three zones. Fibroblasts take up more than 90% of the cells in zone 3. The morphology of fibroblasts is variable across the three zones (Figure 2).

 The structure of periosteum varies with age. In infants and children it is thicker, more vascular, active, and loosely attached as compared to adults where it is thinner, less active, and firmly adherent [8]. In all age groups, the cells of the periosteum retain the ability to differentiate into fibroblasts, osteoblasts, chondrocytes, adipocytes, and skeletal myocytes. The tissues produced by these cells include cementum with periodontal ligament fibers and bone. The periosteum has a rich vascular plexus and is regarded as the “umbilical cord of bone” [9]. The vasculature system of the periosteum was first studied in detail by Zucman and later by Eyre-Brook [10].Bourke's studies showed that the capillaries supplying blood to bone reside within the cortex linking the medullary and periosteal vessels; a recent study has even shown that periosteal cells release vascular endothelial growth factor [11] which promote revascularization during wound healing. Recently, studies have reported the existence of osteogenic progenitors, similar to mesenchymal stem cells (MSCs), in the periosteum [12, 13]. Under the appropriate culture conditions, periosteal cells secrete extracellular matrix and form a membranous structure [14]. The periosteum can be easily harvested from the patient's own oral cavity, where the resulting donor site wound is invisible. Owing to the above reasons, the periosteum offers a rich cell source for bone tissue engineering; hence, the regenerative potential of periosteum is immense.

## 3. Periosteum as a Tool in Medicine and Dentistry

Developing bone substitutes for bone defect repair has inspired orthopedic surgeons, bone biologists, bioengineering researchers to work together in order to design and develop the promising products for clinical applications. Duhame in the year 1742 can be considered the first investigator to study the osteogenic potential of periosteum and published his findings in the article “Sur le Development et la Crueded Os des Animaux” [15]. A century later, another French surgeon, Ollier, discovered that the transplanted periosteum could induce de novo bone formation. One of the earliest experimental studies to demonstrate osteogenic potential of periosteum was that of Urist and McLean who reported that periosteum produced bone when transplanted to the anterior chamber of the eye of the rat [16]. Skoog subsequently introduced the use of periosteal flaps for closure of maxillary cleft defects in humans; he reported the presence of new bone in cleft defects within 3–6 months following surgery [17]. Since then, surgeons have reported the successful use of maxillary periosteal flaps [18, 19] as well as periosteal grafts from the tibia or rib. Melcher observed that new bone is laid down in parietal bone defects of rats and was deposited by periosteum that had not been previously elevated or disturbed in any other way [20], while other investigators have suggested that the contact between the periosteal flap or graft and the underlying bone is crucial to stimulation of osteogenesis [21, 22]. More recently, the osteogenic/chondrogenic capacity of periosteum and related mechanisms have been confirmed, and the underlying biology is better understood through a number of studies [23–40].

**Figure 3:**
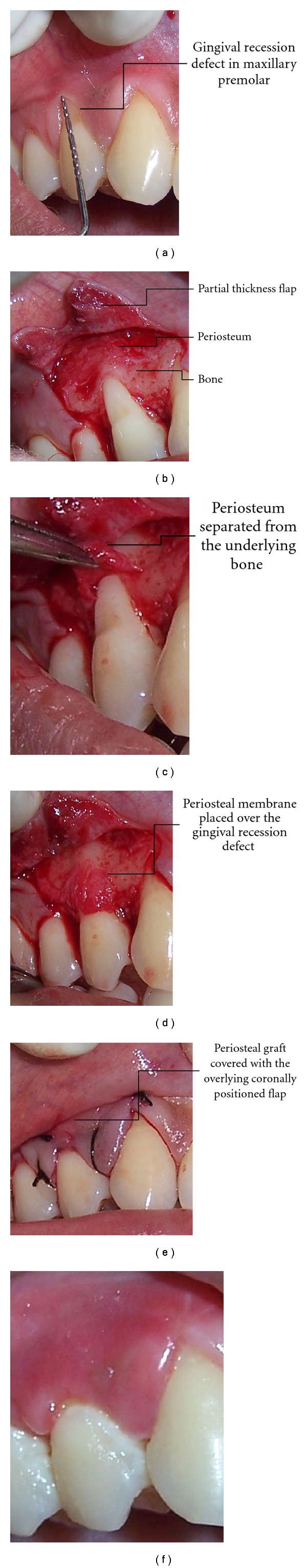
The use of periosteum for the treatment of gingival recession defect. (a) Clinical photograph showing gingival recession defect in relation to the maxillary first right premolar. (b) A partial thickness flap lifted to expose the underlying periosteum covering the alveolar bone. (c) The periosteum which is separated from the underlying bone. (d) The periosteum is used as a pedicle graft for covering the recession defect. (e) The periosteal graft is covered with the overlying coronally advanced flap which is sutured using 4–0 silk suture. (f) Satisfactory treatment outcome.

 The use of periosteum in dentistry is not new. Various research papers have been published explaining the osteogenic potential of human periosteal grafts [41, 42]. The use of periosteum as a GTR has been suggested by many studies [43–46], although long-term results are still awaited to establish the regular and the most effective use of periosteal grafts as barrier membranes. The need for a graft, which has its own blood supply, which can be harvested adjacent to the recession defect in sufficient amounts without requiring any second surgical site and has a potential of promoting the regeneration of lost periodontal tissue is a long-felt need. The adult human periosteum is highly vascular and is known to contain fibroblasts and their progenitor cells, osteoblasts and their progenitor cells, and stem cells. In all the age groups, the cells of the periosteum retain the ability to differentiate into fibroblasts, osteoblasts, chondrocytes, adipocytes, and skeletal myocytes. The tissues produced by these cells include cementum with periodontal ligament fibers and bone; in addition the presence of periosteum adjacent to the gingival recession defects in sufficient amounts make it a suitable graft. Recent papers published have shown promising results with the use of periosteum in the treatment of gingival recession defects (Figure 3) [47, 48]; moreover, with the advancement in tissue engineering techniques the periosteal derived stem cells have been grown effectively to reconstruct lost tissues. Periosteum-derived progenitor cells may serve as an optimal cell source for tissue engineering based on their accessibility, ability to proliferate rapidly, and capability to differentiate into multiple mesenchymal lineages. The periosteum is a specialized connective tissue that forms a fibrovascular membrane covering all bone surfaces except for that of articular cartilage, muscle, and tendon insertions and sesamoid bones. Cells residing within the periosteum may be excised from any number of surgically accessible bone surfaces; in addition, when properly stimulated, the periosteum has the potential to serve as a bioreactor supporting a dramatic increase in the progenitor cell population over the course of a few days. Further, once the cells are removed from the periosteum, they have the potential to proliferate at much higher rates than bone marrow, cortical bone, or trabecular bone-derived progenitor cells [49].

 In addition to their robust proliferation aptitude, it is well established that periosteum-derived progenitor cells have the potential to differentiate into both bone and cartilage. Further, their potential for regenerating both bone and cartilage constructs is superior to that of adipose-derived progenitor cells and comparable with that of bone marrow-derived mesenchymal stem cells. A recent study by De Bari et al. indicates that periosteal progenitor cells are able to differentiate not only into bone and cartilage cells but also into adipocyte and skeletal myocyte cells [50]. There is a growing requirement for dentists to regenerate alveolar bone as a regenerative therapy for periodontitis and in implant dentistry. Concerning the donor site, it is easier for general dentists to harvest periosteum than marrow stromal cells, because they can access the mandibular periosteum during routine oral surgery [51]; also the regenerative potential of periosteum has been effectively used in “osteodistraction” which has the benefit of simultaneously increasing the bone length and the volume of surrounding tissues. Although distraction technology has been used mainly in the field of orthopedics, early results in humans  indicated that the process can be applied to correct deformities of the jaw. These techniques are now utilized extensively by maxillofacial surgeons for the correction of micrognathia, midface, and fronto-orbital hypoplasia in patients with craniofacial deformities [52].

## 4. Conclusion

The use of periosteum can revolutionize the success of various dental treatments which require either bone or soft tissue regeneration; particularly the future use of periosteum must be explored in periodontal and implant surgical procedures. Although the regenerative potential of periosteum has been proved by numerous studies, till date the use of periosteum-derived grafts has still not become a standard tool in the armamentarium of dental surgeons, and it may still need some time, and further research before the full regenerative potential of periosteum is utilized.
